# Relationship between seasons and postpartum depression: A systematic review and meta‐analysis of cohort studies

**DOI:** 10.1002/brb3.2583

**Published:** 2022-05-03

**Authors:** Tao‐Hsin Tung, Dina Jiesisibieke, Qinyi Xu, Yen‐Ching Chuang, Zhu Liduzi Jiesisibieke

**Affiliations:** ^1^ Evidence‐based Medicine Center Taizhou Hospital of Zhejiang Province affiliated to Wenzhou Medical University Taizhou China; ^2^ Department of Neurology Taizhou Hospital of Zhejiang Province affiliated to Wenzhou Medical University Taizhou China; ^3^ Peking University Third Hospital Beijing 100191 China; ^4^ Institute for Hospital Management Tsing Hua University Shenzhen Campus China; ^5^ Institute of Public Health & Emergency Management Taizhou University Taizhou Zhejiang China; ^6^ Business College Taizhou University Taizhou Zhejiang China

**Keywords:** meta‐analysis, postpartum depression, season

## Abstract

**Objective:**

As the reproduction season's effect on the mental health status is unknown, this study aims to explore the association between seasons and postpartum depression.

**Methods:**

A comprehensive search in databases, including PubMed, Cochrane Library, and EMBASE, was performed to identify studies reporting the relationship between reproduction season and postpartum depression. The latter was assessed using certain methods. Moreover, the study design and duration, sample size, the definition of four seasons, outcome assessment, method, and conclusion were extracted. Two independent authors screened the studies independently, and PRISMA 2020 was used as the reporting standard (PRISMA registration ID is 284524).

**Results:**

A total of five studies including 103,986 participants met our criteria. In the sensitivity analysis, the result of the meta‐analysis shows that women who gave birth in spring, summer, or autumn had a lower risk of postpartum depression compared to those who gave birth in winter (RR: 0.83; 95% confidence interval [CI]: 0.78–0.88).

**Conclusion:**

Women who gave birth in the other seasons were less likely to have postpartum depression compared to those who gave birth in winter. This result could help couples make overall decisions and help the puerpera take preventive measures against postpartum depression.

## Abstract 2

Women who gave birth in the other seasons were less likely to have postpartum depression compared to those who gave birth in winter. This result could help couples make overall decisions and help the puerpera take preventive measures against postpartum depression.

## 1 INTRODUCTION

Postpartum depression (PPD) is typically identified as a one‐time depressed mood and decreased interest or enjoyment in activities during the postpartum period, which could affect 15% of women after childbirth (American Psychiatric Association, 2013; Corral et al., 2007; Yu et al., 2021). Regarding diagnosis, according to the Diagnostic and Statistical Manual of Mental Disorders Fifth Edition (DSM‐5), PPD is characterized by major depression that occurs within 4 weeks after childbirth (American Psychiatric Association, 2013). However, in a clinical setting, it may occur between 4 weeks and 12 months after delivery (Stewart & Vigod, 2016). Although there is some uncertainty, the poor influence of women (Brummelte & Galea, 2016), their family (Simhi et al., 2021) and relationships with children (Letourneau et al., 2017), and society (Ongeri et al., 2018) is certain.

PPD can cause serious harm to women and their families. Studies suggest that more than a quarter of women have a history of PPD and suffer redevelopment after giving birth. Depressed mothers may respond poorly to their babies, which may harm their attachment. Many other serious problems occur during this period. For example, infants with mothers suffering from PPD will have an increased risk of sudden infant mortality, cognitive impairment, and behavior disorders at key stages of their development. Therefore, early detection and intervention of PPD are important measures for ensuring the quality of life of postpartum mothers and infants (Slomian et al., 2019).

Potential seasonal trends of PPD are important and the result is unknown. Seasonal factors have been suggested in some studies during the postpartum period, reporting more cases of PPD in autumn and winter than in summer (Sit et al., 2011; Yang et al., 2011). However, Henriksson et al. found that there was no consistent seasonal pattern in depressive symptoms (Henriksson et al., 2017). These contradictory results might be explained by differences in geographical location, climate, and assessment method. Consequently, we systematically reviewed the literature to determine whether existing data support the hypothesis of seasonal variation in the prevalence and symptoms of postpartum depression. Having knowledge about how depressive symptoms develop during the postpartum period according to seasons would contribute to a better understanding of risk factors, mechanisms, and epidemiology of postpartum depression. If seasonality can affect PPD, then early detection and treatment could be used, which could improve the life quality of the puerpera and assist couples in making pregnancy decisions.

The study aims to show the association between seasonal variation and PPD, draw attention to seasonal patterns, identify the vulnerable time of delivery, and propose early detection and interventions to prevent or minimize the risk of developing PPD.

## 2 MATERIALS AND METHODS

### 2.1 Literature search

**FIGURE 1:**
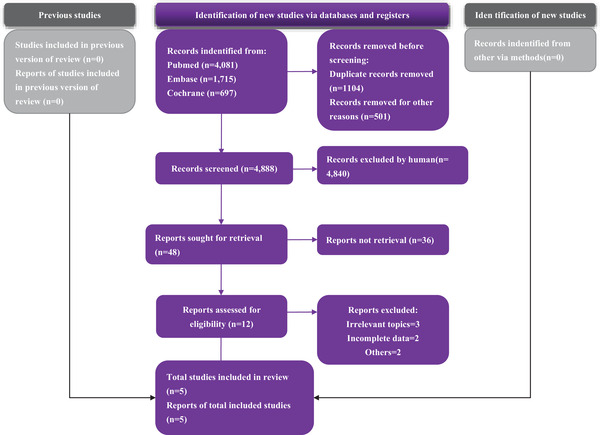
Preferred Reporting Items for Systematic Reviews and Meta‐Analyses (PRISMA) study flow chart

We performed a literature search using PubMed, Cochrane Library, and EMBASE for all related papers published until October 20, 2021 without any language restriction. The search strategy was “season, weather, month, temperature, environment, humidity, or climate as well as postpartum depression, postnatal depression, or post‐natal depression.” We also checked the list of similar articles. This study follows the Preferred Reporting Items for Systematic Reviews and Meta‐Analyses (PRISMA) guidelines (Figure 1). PRISMA 2020 were served as the reporting norm (PRISMA registration ID is 284524).

### 2.2 Study selection

The inclusive criteria are as follows: (1) The outcome should be postpartum depression, and its measurement should be clarified by either an assessment questionnaire or hospital records; (2) information about the four seasons should be clarified; (3) the measurement point should be clearly presented; and (4) the study design should be cohort study. We excluded those with depression before giving birth. Headings and/or summaries of research results were scanned, and the full text was obtained when a study appeared to meet the above criteria. To determine if any information was related, we looked at the complete text. We examined headings and summaries of the research papers and reviewed the full text when they met the above‐mentioned inclusion criteria. We carried out a detailed examination to determine whether the information was potentially linked. Dina Jiesisibieke, Qinyi Xu and Zhu Liduzi Jiesisibieke independently completed the selection procedure, and the disagreements were resolved by discussion with a third principal co‐author (Prof. Tao Hsin Tung).

### 2.3 Data extraction and quality assessment

We extracted the following information: first author, country, publication year, database used, study design, study duration, sample size, the definition of four seasons, outcome assessment method, and conclusion. The Newcastle–Ottawa Scale (NOS) was applied to assess the quality of the included studies. The NOS application has three aspects: study group selection, comparability, and outcome evaluation, which were used to assess the quality of the cohort studies (Chi et al., 2017). For each element of the selection and result fields, we can assign up to one star and, for reasons of comparability, up to two stars. Seven or more stars (*) lead to high‐quality research. Furthermore, to improve the reproducibility and comparability of this subject with future examinations of a similar theme, we also included a risk of bias assessment using Risk of Bias in Nonrandomized studies of Interventions (ROBINS‐I) (Table 1).

**TABLE 1:** Characteristics of the included studies

No.	Study, year, country, database used	Study design	Study duration	Sample size	Definition of season	Outcome assessment method	Outcomes	NOS score
1	Jewell (Jewell et al., 2010), 2010, USA, PubMed	longitudinal study	2 years	67079 women	Sp: Apr–Jun Su: July–Sep F: Oct–Dec W: Jan–Mar	Pregnancy Risk Assessment Monitoring System (PRAMS)	No significant relationship was found between mild or moderate PPD and either season of birth or length of daylight at birth.	S: ** C: ** O: ***
2	Henriksson (Henriksson et al., 2017), 2017, Sweden, PubMed	longitudinal population‐based study	6 years	4085 women	Sp: Jan–Mar Su: Apr–Jun F: July–Sep W: Oct–Dec	Edinburgh Postnatal Depression Scale (EPDS)	Women who gave birth in winter had an increased odds of depressive symptoms at 6 weeks postpartum.	S: *** C: * O: ***
3	Sit (Sit et al., 2011), 2010, USA, PubMed	longitudinal study	5 years	9339 women	Months	Edinburgh Postnatal Depression Scale (EPDS)	PPD risk varied significantly across 12‐months—risk was highest in December.	S: **** C: ** O: ***
4	Yang (Yang et al., 2011), 2010, China (Taiwan), PubMed	longitudinal study	4 years	2107 mothers	Sp: Mar–May Su: Jun–Aug F: Sep–Nov W: Dec–Feb	Edinburgh Postnatal Depression Scale (EPDS)	The risk of PD for winter deliveries were higher compared to other seasons.	S: *** C: ** O: ***
5	Chan (Chan et al., 2019), 2019, New Zealand, PubMed	A secondary analysis of data from a prospective cohort study	37 weeks	260 women	Sp: Sept–Nov Su: Dec–Feb F: Mar–May Wr: Jun–Aug	Recorded diagnosis (ICD code)	Prevalence was significantly higher in winter and spring antenatally and in spring postnatally compared to autumn.	S: *** C: * O: ****

*Note*: Scale domains: S, selection of study group; C, comparability; O, outcome assessment.

Abbreviations: NOS, Newcastle–Ottawa Scale; PPD, postpartum depression.

### 2.4 Statistical analysis

In this study, Review Manager 5.3 was used. We presented the odds of PPD as odds ratio (OR) with 95% confidence interval (CI) and assessed heterogeneity using the *I* ^2^ statistic. This evaluates the degree of variation across studies by heterogeneity rather than only chance. An *I* ^2^ value of 50% or more represents substantial heterogeneity (Higgins, 2003). Since the sample size of Jewell et al. was relatively larger than that of other studies and significantly impacted the result (Jewell et al., 2010), we removed the study and conducted sensitivity analysis. Besides subgroup analysis, we also combined the data of spring, summer, and autumn together, and then we found that *I* ^2^ was 71%, then we used a random‐effect model. We therefore conducted a sensitivity analysis and found that *I* ^2^ was 26%; then, we applied a fixed‐effect model.

## 3 RESULTS

### 3.1 Characteristics of included studies

In this study, 1096 papers were identified after the elimination of duplication (Figure 1). Finally, five publications reporting 103,986 participants satisfied the predetermined inclusion criteria. These studies were published between 2010 and 2019. All the included studies are rated more than seven stars in the NOS assessment. The studies by Henriksson et al., Yang et al. and Sit et al. applied Edinburgh Postnatal Depression Scale (EPDS) to assess depression (Sit et al., 2011; Yang et al., 2011). Jewell et al. used Pregnancy Risk Assessment Monitoring System (PRAMS) to assess PPD (Jewell et al., 2010). Chan et al. included recorded diagnoses from hospitals (Chan et al., 2019) (Table 2).

### 3.2 Associations between reproductive season and PPD

**FIGURE 2:**
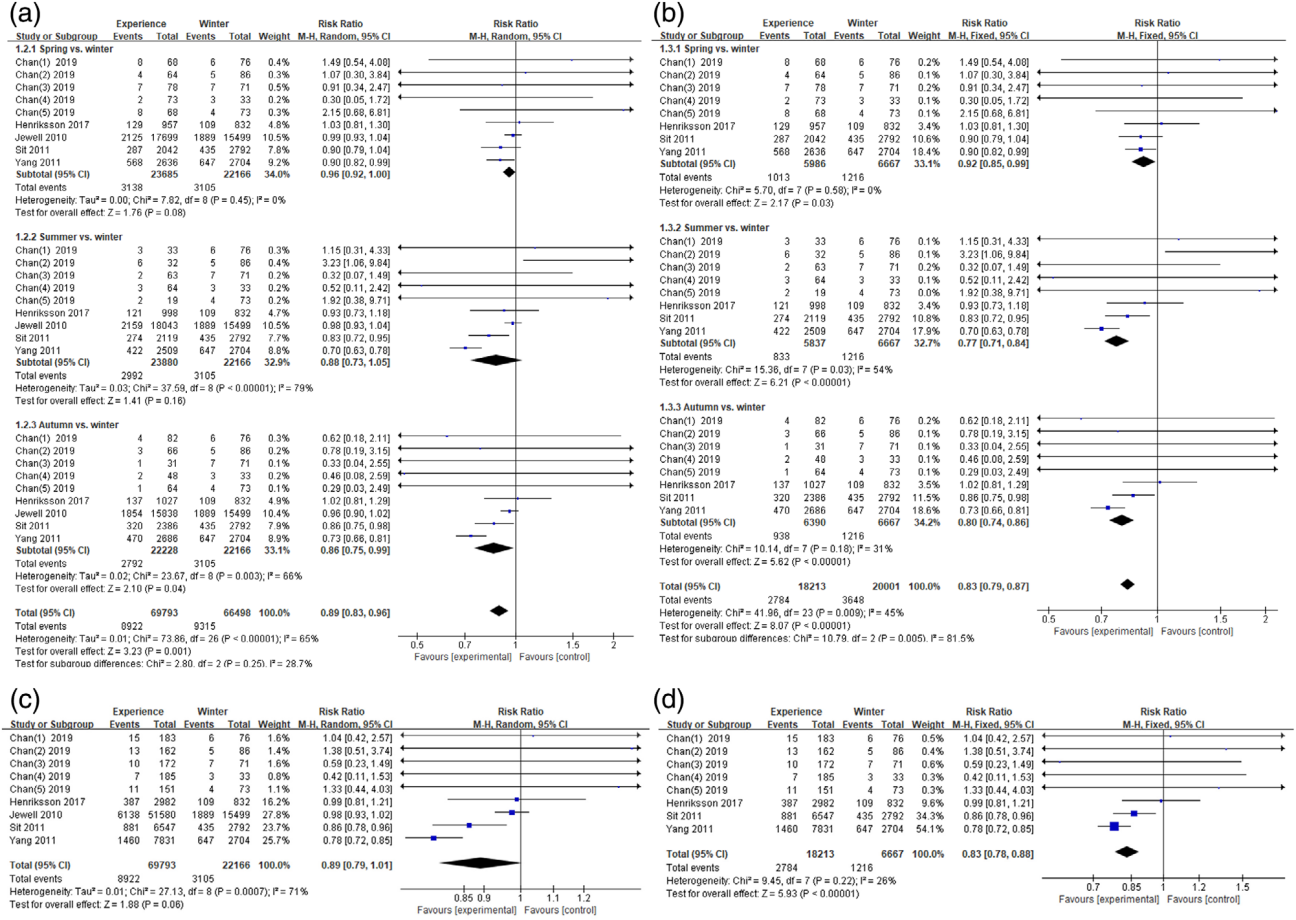
(a) Risk ratio of postpartum depression (PPD) in spring, summer, and autumn compared to winter. (b) Sensitivity analysis of risk of PPD in spring, summer and autumn compared to winter. (c) Risk ratio of PPD in combination of spring, summer and autumn compared to winter. (d) Sensitivity analysis of risk of PPD in the combination of spring, summer and autumn compared to winter. Abbreviations: CI, confidence interval; SE, standard error

A total of six studies provided data for this outcome (Figure 2). Chan et al. (2019) did not find any significant difference between spring, summer, and autumn compared to winter. They found that prevalence was significantly higher for spring than for winter (Chan et al., 2019). In their study, Henriksson et al. included 129, 998, and 1027 women who delivered in spring, summer, and autumn, respectively, along with 832 women who delivered in winter, and the authors generally found no consistent seasonal patterns regarding PPD (Henriksson et al., 2017). Sit et al. included 2042, 2119, 2386, and 2792 women who delivered in spring, summer, autumn, and winter, respectively, and found that prevalence of PPD is high in winter compared to other seasons (Sit et al., 2011). Yang et al. included 2636, 2509, 2686, and 2704 women who delivered in spring, summer, autumn, and winter, respectively, and the authors also found that PPD is high in winter compared to the other seasons (Yang et al., 2011). Jewell et al. included 17,699, 18,043, 15,838, and 15,499 women who delivered in spring, summer, autumn, and winter, respectively, and found no significant incidence of PPD among four seasons (Jewell et al., 2010). The result showed that delivering in spring (RR: 0.96; 95% CI: 0.92–1.00) or summer (RR: 0.88; 95% CI: 0.73–1.05) was not significantly associated with lower risk of PPD compared with delivering in winter. However, delivering in autumn was significantly associated with lower risk of PPD compared with delivering in winter (RR: 0.86; 95% CI: 0.75–0.99). Since the sample size of one included study (Jewell et al., 2010) was relatively larger than other findings, we further performed a sensitivity analysis and found that risks of PPD were significantly associated with spring (RR: 0.92, 95% CI: 0.85–0.99), summer (RR: 0.77, 95% CI: 0.71–0.84), and autumn (RR: 0.80, 95% CI: 0.74–0.86) (Figure 2a,b).

Since we would like to investigate the combination of spring, summer, and autumn versus winter associated with PPD, we calculated the total number of spring, summer, and winter. We also found a significant association (RR: 0.83; 95% CI: 0.78–0.88) by the sensitivity analysis (Figure 2c,d).

### 3.3 Publication bias

**FIGURE 3:**
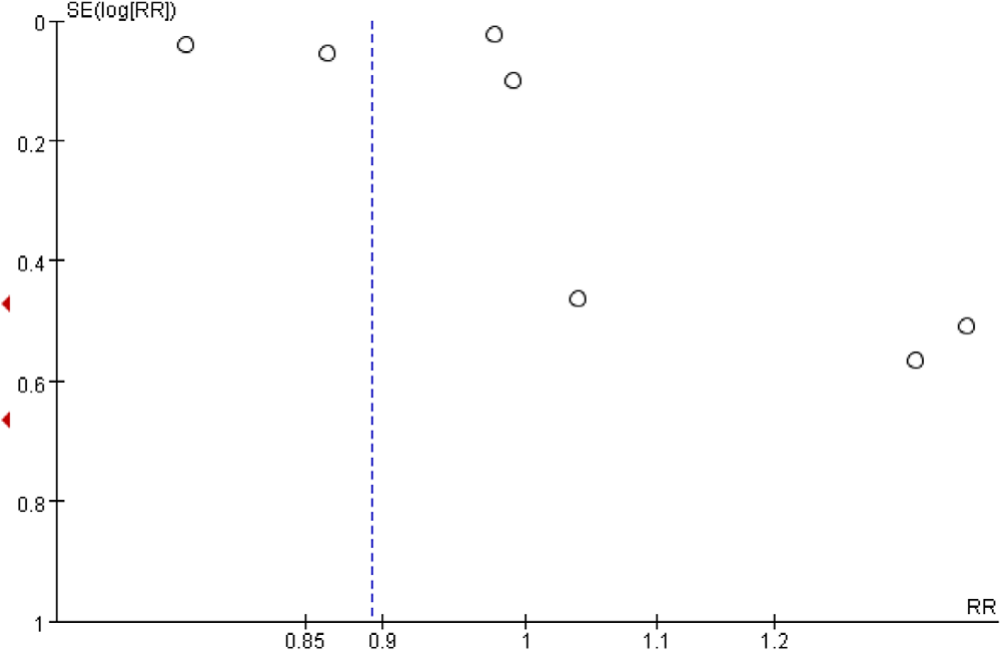
Funnel plot of the included studies

Publication bias was assessed using a funnel plot (Figure 3). It referred to the publication of studies depending on the direction and statistical significance of the results, and the first systematic investigations of publication bias focused on this aspect of the problem (Kuo et al., 2021). In our study, the funnel graph was asymmetric, indicating a degree of publication bias in this study.

### 3.4 GRADE summary of findings table

A summary of findings and evaluation of GRADE for each outcome is presented in Table 3. The quality of evidence from the included studies was considered moderate overall.

**TABLE 2:** GRADE summary of findings

Risk of postpartum season having depression						
Patient or population: Postpartum women Setting: USA, Sweden, China, and New Zealand Intervention: Combination of spring, summer, and autumn Comparison: Winter						
	Anticipated absolute effects* (95% CI)					
Outcomes	Risk in control	Risk in experiment	Relative effect (95% CI)	No. of participants (studies)	Qualityof the evidence(GRADE)	Comments
Risk of depression	182 per 1000	153 per 1000	RR: 0.83 (0.78–0.88)	28,880	⨁⨁⨁◯ Moderate	NA

*Notes*: GRADE Working Group grades of evidence. High quality: We are very confident that the true effect lies close to its estimate. Moderate quality: We are moderately confident regarding the effect estimate: The true effect is likely to be close to the estimate of the effect, but there is a possibility that it is substantially different. Low quality: Our confidence in the effect estimate is limited. The true effect may be substantially different from the estimate. Very low quality: We have little confidence in the effect estimate: The true effect is likely to be substantially different from the estimate.

Abbreviation: CI, confidence interval; RR, risk ratio.

* The risk in the intervention group (and its 95% CI) is based on the assumed risk in the comparison group and the relative effect of the intervention (and its 95% CI).

**TABLE 3:** Risk of bias assessment using ROBINS‐I

		Preintervention		At intervention		Postintervention			Total
Author	Types of research	Bias due to confounding	Bias in selection of participants into study	Bias in classification of interventions	Bias due to deviations from intended interventions	Bias due to missing data	Bias in measurement of outcomes	Bias in selection of the reported outcomes	Total bias
Jewell et al. (2010)	longitudinal study	High risk	High risk	Low risk	Moderate risk	Low risk	Moderate risk	Low risk	High risk
Henriksson et al. (2017)	longitudinal study	Moderate risk	High risk	Low risk	High risk	Low risk	Moderate risk	Low risk	High risk
Sit et al. (2010)	longitudinal study	Moderate risk	Low risk	Low risk	Low risk	Low risk	Low risk	Low risk	Low risk
Yang et al. (2010)	longitudinal study	Moderate risk	Low risk	Moderate risk	Low risk	Low risk	Low risk	Low risk	Moderate risk
Chan et al. (2019)	prospective cohort study	Moderate risk	Moderate risk	Low risk	Low risk	Low risk	Moderate risk	Low risk	Moderate risk

## 4 DISCUSSION

### 4.1 Clinical implications

The evaluation results of five publications included in this study provide evidence of an association between seasonal changes and PPD. Our results, together with those of other studies, show that women who delivered in winter have increased odds of PPD compared with those who delivered in the other three seasons. The results provide further evidence for the effect of seasonal patterns in PPD.

Some factors could be considered to explain the higher chances of PPD during winter, although the seasonal mechanism underlying PPD is not clear. Shorter daytime is one of the most obvious differences between winter and other seasons, which may provide a possible explanation for the variation (Yan et al., 2019). Hormone levels in the body play a major role in depression and change with the influence of season and light (Melrose, 2015). Being exposed to high intensity light is an important factor of the proper working of circadian system as well as well‐being (Bilu et al., 2020). Srinivasan et al. suggested the melatonin levels as a possible explanation for the correlation between shorter daytime in winter with an increased risk of depression (Srinivasan et al., 2006). Blume et al. also noted the association between reduced day‐length, which may cause fewer outdoor activities and less exposure to light, and an increased potential of the serotonin transporter may correlate with depressive mood (Blume et al., 2019). It has been suggested that postpartum mothers who suffer from rapid hormonal changes have a greater risk of developing depression (Nguyen et al., 2019). Insufficient social support can highly increase the risk, so staying indoors, having fewer outdoor activities, and having connections with other people during winter may reduce the feelings of support needed by the postpartum mother (Kim et al., 2014).

Early postpartum follow‐up and professional intervention for high‐risk groups, including psychological reports, postpartum courses, and continuous care model, often show better preventive effects (Dennis, 2005). As PPD develops in a limited time, after delivering, regular contact with their health care providers helps detect depressive symptoms and handle these problems in time during the PPD (Mukherjee et al., 2018). Considering the importance of early detection and intervention, there has been significant interest in the positive effect of identifying high‐risk mothers delivering in winter to increase the prevention of PPD and save a large medical expenditure by researchers across the world. A meta‐analysis also proved that walking could help the women to overcome PPD (Pentland et al., 2021). Our study also suggests that mothers delivering in winter may benefit from postpartum follow‐up and support from the care providers and other social contacts, which may promote the long‐term well‐being of mothers and babies.

### 4.2 Clinical practice

The result of PPD affects the mother and her ability to care for children, especially in less privileged countries (Asare et al., 2021). The influence of the maternal environment and phenotype on that of the offspring can allow mothers to fine‐tune its development trajectory and the resulting phenotype, sometimes long after the offspring has reached independence (Bebbington & Groothuis, 2021). Our study suggests that decision‐making for the delivery season is important before labor. Understanding the association between birth season and PPD could help in investigating the causes of PPD, and the couples could make better birth decisions and prevent the incidence of PPD in their family.

### 4.3 Heterogeneity of meta‐analysis

Within the meta‐analysis, there may be heterogeneity if the population risk sample estimates were of different amplitudes (Sedgwick, 2015). Statistic *I* ^2^ implies the percentage change between the selected studies which is due to heterogeneity rather than random. In this study, we used the random effect model when total *I* ^2^ statistics were 63%. In the sensitivity analysis, we excluded Jewell's study and the *I* ^2^ decreased to 45%. Therefore, in our study, this problem was caused by the different magnitude of various studies.

### 4.4 Methodological considerations

There were some limitations in the study. First, additional factors showing the influence on depression such as outdoor activities, social support, or exposure to violence were not evaluated. Second, some additional differences between the studies may cause the results of higher odds of PPD in winter. For example, the people in the study were distributed at different latitudes, where the days and climate could be different, even though the seasons were the same for each group. The publications included in the review were also different in depression assessment methods, sample sizes, population characteristics, and statistical treatments. Finally, our study was unable to demonstrate the association between PPD and delivering season at the statistic level. Future studies should consider other full effects of different seasons in a broader perspective, so the influence of seasonal variations on PPD can be understood and handled more scientifically by stakeholders including the care providers, health policymakers, family members, and more importantly, the mother.

## 5 CONCLUSION

In conclusion, women who gave birth in spring, summer, or autumn were less at risk for PPD than those who gave birth in winter. This result could help couples make comprehensive decisions and assist the puerpera to take preventive measures against PPD.

## CONFLICT OF INTEREST

The authors declare no conflict of interest.

## AUTHOR CONTRIBUTIONS

Tao‐Hsin Tung, Dina Jiesisibieke, Qinyi Xu, Yen‐Ching Chuang, and Zhu Liduzi Jiesisibieke conducted the study and drafted the manuscript. All the authors participated in the design and performed data synthesis. Tao‐Hsin Tung and Zhu Liduzi Jiesisibieke conceived the study and participated in its design and coordination. All the authors read and approved the final manuscript.

### PEER REVIEW

The peer review history for this article is available at https://publons.com/publon/10.1002/brb3.2583.

## FUNDING INFORMATION

None.

## Data Availability

All data underlying the findings are within the paper.
